# Photobiomodulation therapy as an adjunct to resistance exercises on muscle metrics, functional balance, functional capacity, and physical performance among older adults: A systematic scoping review

**DOI:** 10.1007/s10103-024-04177-x

**Published:** 2024-09-03

**Authors:** Prabal Kumar, Shashikiran Umakanth, Girish N

**Affiliations:** 1 Department of Physiotherapy, Manipal College of Health Professions, Manipal Academy of Higher Education, Manipal, Karnataka India; 2 Department of Medicine, Dr. TMA Pai Hospital, Manipal Academy of Higher Education, Udupi, Karnataka India

**Keywords:** LASER, Older adults, Photobiomodulation, Resistance exercise, Sarcopenia

## Abstract

This scoping review aims to summarize the literature on photobiomodulation (PBM) therapy as an adjunct to resistance exercise among older adults, focusing on its effects on muscle metrics, functional balance, capacity, and physical performance. The participants included were older adults aged ≥ 60 years and either gender. The concept was the application of PBM and resistance exercises with no limits on the context parameters. The databases Medline, Embase, Scopus and Web of Science were searched from inception till February 2024. Methodological quality was assessed using the Cochrane risk of bias 2.0. A total of 10 studies were included in the review. PBM, along with resistance training, was reported in six studies, whereas four reported PBM with isometric fatigue protocol. PBM with a wavelength of 808 nm was given on the belly of the rectus femoris muscle in most of the studies. The common dosimetry parameters used were- power density 35.7 W/cm^2^, energy density 250 J/cm^2^, 8 sites, energy per site 7 J, duration of 70 s per site, spot size per diode of 0.028 cm^2^, and stationary contact of application. The application of PBM therapy alongside resistance exercise has been found to improve muscle metrics, functional capacity, and functional performance and reduce fatigability when compared with the control group in most of the studies. When incorporating PBM therapy as an adjunct to resistance training, it is crucial to consider the dosimetry parameters involved—to achieve the intended therapeutic effect. Adjusting treatment parameters with this information can optimize the effectiveness of PBM therapy and improve treatment outcomes for patients.

**Supplementary information:**

The online version contains supplementary material available at 10.1007/s10103-024-04177-x.

## Introduction

Population ageing is a global phenomenon affecting social demographics [1]. As the person ages, there are various evident changes in the musculoskeletal system [2]. The changes in the muscle metrics, including the decrease in muscle mass, muscle strength, and physical performance, have been extensively studied in the literature [3]. These changes signify a prevalent geriatric disorder with significant healthcare costs [4], identified as sarcopenia by Rosenberg in 1997 [5]. Sarcopenia was recognized as a distinct clinical entity in 2016 within the International Classification of Diseases, Tenth Revision, Clinical Modification ICD-10-CM code (M62.84) [6]. Managing sarcopenia is crucial, and literature provides evidence for a multi-component program with resistance exercises as the cornerstone [7]. Over the past decade, researchers have begun to incorporate photobiomodulation (PBM), a non-invasive therapy that uses light to stimulate cells and tissues, as a therapeutic intervention to explore its positive impact on the musculoskeletal system [8].

Scientific literature has identified several key benefits of PBM therapy, including enhanced performance [8], reduced fatigue [9], increased strength [10] and improved relaxation [11, 12]. This is largely attributed to the biomodulatory effect of light, which is absorbed by chromophores and transformed into chemical energy that triggers biological responses locally or systemically within the organism [13]. Likewise, mitochondria absorb wavelengths in the red and infrared spectrum and play a crucial role in accelerating the synthesis of adenosine triphosphate (ATP), which is the primary source of cellular energy [14, 15]. It is hypothesized that optimizing ATP synthesis resources may have a positive impact on functional performance, given that muscle activity requires a significant amount of energy expenditure [8]. Resistance exercise training also appears to augment the respiratory capacity and intrinsic function of skeletal muscle [16]. This implies that the application of PBM therapy as an adjunct intervention to resistance training could combat sarcopenia.

The selection of appropriate dosimetric parameters by the therapist is a crucial factor in achieving desired outcomes with PBM therapy [17]. The literature recommending the dosimetry of PBM therapy to enhance exercise performance is limited to young healthy adults [18]. The non-invasive and positive effect of the PBM therapy application among young adults has given clinicians a novel approach to using PBM therapy among older adults [17, 18]. However, there is ambiguity on treatment parameters, outcome measures, and the benefits of utilizing PBM therapy as a therapeutic adjunct to resistance exercise for muscle metrics in older adults. The review would be a valuable resource for physicians and researchers who intend to utilise PBM in research studies and clinical practice as an adjunct to resistance exercise for managing sarcopenia. Therefore, the hypothesis is that incorporating PBM therapy alongside resistance exercise would further enhance muscle function, physical performance, functional balance, and reduce fatigue in older adults. Hence, the aim of this systematic scoping review was to identify and summarize the currently available literature on dosimetric parameters of PBM, parameters of resistance exercise and the effect of adjunct PBM along with resistance exercise on muscle metrics, functional balance, functional capacity, and physical performance among older adults. Considering the aforementioned contextual background, the objective of this study is to examine the following questions:

1. What are the most commonly used dosimetric parameters (wavelength, dose, number of points, place of application)?
2. What are the most commonly used resistance exercise parameters (frequency, intensity, time/session, total duration, equipment used)?
3. What change in muscle metrics (1-Repition Maximum (1-RM), peak torque, muscle thickness), functional balance, functional capacity, physical performance, and fatigue was observed following PBM and/ or resistance exercise training?

## Methods

The scoping review methodology was chosen as it is the most appropriate method to identify the key characteristics related to the topic of investigation. This review followed the Joanna Briggs Institute (JBI) Scoping Review Methodology [19] and Preferred Reporting Items for Systematic reviews and Meta-Analysis extension for Scoping Reviews (PRISMA-ScR) checklist was used for reporting processes [20].

### Eligibility criteria

The selection criteria incorporated the PCC (Participants, Concept, and Context) format [19]. The participants included older adults aged ≥ 60 years, either gender. The concept was the implementation of PBM and resistance exercise with muscle metrics (1-RM, peak torque, muscle thickness), functional balance, functional capacity, physical performance, and fatigue as an outcome measure. We set no limits on the context parameters, and all the settings were considered. This scoping review included all the relevant original research literature (published and ongoing), irrespective of the study designs that used PBM and resistance exercise among older adults. It consisted of the implementation of active or sham PBM with resistance training. The studies were excluded if they failed to meet the inclusion criteria and/ or: (a) not in English language (no funding was available to allow for the translation process), (b) no extractable data (studies did not report the dosimetry parameters of PBM and/ or exercise training parameters), (c) full-text not available, (d) if any additional nutritional supplement is given, (e) abstract and conference proceedings, and (f) animal studies.

### Information sources and search strategy

In this systematic scoping review, the researchers employed a comprehensive search strategy across four electronic databases: Medline (PubMed), Embase, Scopus and Web of Science from inception till February 2024. The search was carried out after the validation of the keywords by the expert. The keywords related to PBM, older adults, and resistance exercises were used to make search strings in the databases. The search strategy was: (older adults OR older person OR aged) AND (photobiomodulation OR low-level laser therapy OR phototherapy OR LASER therapy) AND (resistance exercise OR strengthening OR exercise). The detailed search strategy is attached as supplementary material 1.

### Selection of sources of evidence

Two reviewers, PK and GN, independently searched the literature. The identified studies were imported to Rayyan (Ref. # 933536) software. After resolving the duplicates, two reviewers (PK and GN) conducted a separate title and abstract screening. If the study was deemed suitable, it progressed to retrieving the full text. Disagreements between reviewers were resolved by consensus, and a third independent expert (SU) was approached if disagreement could not be resolved in discussion. PK did a full-text reading, and data extraction was carried out from the relevant studies.

### Data collection process and data items

Two included studies were randomly selected and shared with all the reviewers, and the data charting sheet was prepared individually and piloted. Further, the data charting sheet was finalized after a consensus discussion with all the authors. The data extracted for PBM therapy parameters included [wavelength (nm), mode, peak power (mW), power density (W/cm^2^), energy density (J/cm^2^), energy per diode (J), energy per site (J), number of diodes used (cluster probe), treatment time (per site, per session), number of sites (each leg), spot size (per diode, cm^2^), frequency (number of days/week, total number of weeks), treatment deliver (stationary/moving circular, back-n-forth), manufacturer/ brand and model]. For the exercise training components, the following data was extracted [mode of exercise, frequency/week, intensity, time/session, total duration (weeks), initial assessment of intensity, progression, muscle/muscle group targeted, equipment used, supervised/ unsupervised]. Lastly, the data related to change in the outcome measures was extracted [author, year, type of study, demographic characteristics (population, total sample size, number of group, age (year), gender (male/female), outcome measure (active PBM group pre/ post, placebo PBM group pre/ post, control group pre/ post), results, and conclusion]. Data extraction was performed by one reviewer (PK) in a standardized predefined way, and summarized by tabulation (Tables 1 and 2, and 3).

**Table 1:** Photobiomodulation therapy parameters

Authors	Wavelength (nm)	Mode/frequency	Peak power (mW)	Power density (W/cm^2^)	Energy density (J/cm^2^)	Energy per diode (J)	Energy per site (J)	Energy per leg (J)	Number of diodes used (cluster probe)	Treatment time (per site, per session)	Number of sites (per leg)	Spot size (per diode) (cm^2^)	Frequency (no. of days/week, number of weeks)	Treatment delivery (stationary/Moving [circular, back-*n*-forth]) Distance of tissue	Manufacturer/Brand and model
Toma et al., 2013	LASER 808	Continuous	100	12.7	892 each point	NR	7	56	NR	70 s/site	8 Rectus femoris	0.00785	Before fatigue protocol	Stationary in skin contact mode	AsGaAl (Photon Laser III; DMC^®^ São Carlos, SP, Brazil)
Toma et al., 2016	LASER 808	Continuous	100	35.7	250	NR	7	56	NR	70 s/site	8 Quadriceps femoris	0.028	After the strength training	Contact technique	AsGaAl (Photon Lase III; DMC^®^ São Carlos, SP, Brazil)
Matos et al., 2016	LED 638	Continuous	150	NR	11.7	NR	4.5	NR	One diode	20 s	4 Forearm	0.038	Before fatigue protocol	Direct contact with skin surface at 90 degrees	Bios Therapy II, BIOS, São José dos Campos, SP, Brazil)
Vassão et al., 2016	LASER 808	Continuous	100	35.7	250	NR	7	56	NR	70 s/site	8 Rectus femoris	0.028	Before fatigue protocol	Punctual contact technique	AsGaAl laser (Photon Laser III; DMC Importação e Exportação de Equipamentos Ltda, São Carlos, SP, Brazil
Vassão et al., 2018	LASER 808	Continuous	100	35.7	250	NR	7	56	NR	70 s/site	8 Rectus femoris	0.028	Post-exercise session	Punctual contact Technique Vertically	AsGaAl (Photon Laser III; DMC^®^ São Carlos, SP, Brazil)
Fritsch et al., 2019	LASER 850	Continuous	100	3.4	206.9	6	30	240	5 diodes (Cluster probe)	60 s/ site	8 (Vastus lateralis 2 sites; Vastus medialis 3 sites; Rectus femoris 3 sites)	0.029	2/week for 12 weeks Pre-exercise session	Stationary Skin contacts at 90 degrees angle with light skin pressure	Chattanooga Corp., Chattanooga, USA
Tucci et al., 2019	LASER 808	Continuous	100	2	91	NR	4	112	NR	40 s/site	4 Quadriceps femoris	0.05	Post-exercise session	Skin contact mode Perpendicular	DMC Equipment Ltda, São Carlos, Brazil
Rodrigues et al., 2020	LASER 808	Continuous	100	35.7	250	NR	7	56	NR	70 s/site	8 Rectus femoris	0.028	Pre-exercise session	Stationary Skin contact mode	AsGaAl laser (λ: 808 nm) equipment (Therapy XT; DMC, São Carlos, SP, Brazil).
Rodrigues et al., 2022	LASER 808	Continuous	100	35.7	250	NR	7	42	NR	70 s/site	6 (Rectus femoris 3 points; Vastus lateralis 3 points)	0.028	Pre-exercise session	Stationary Skin contact mode	AsGaAl laser (λ = 808 nm) equipment (Therapy XT; DMC São Carlos, São Paulo, Brazil)
Elbanna et al., 2022	LASER 808	Continuous	100	NR	127.39	NR	NR	NR	NR	40 s	2 Calf muscle	0.0314	3 sessions/week for 4 weeks	Punctual contact approach LASER probe at 90 degrees and skin contact	NR

NR not reported; LED Light emitting diode; LLLT Low level laser therapy

**Table 2:** Component of resistance training

Authors	Mode of exercise	Frequency/week	Intensity	Time/ session	Total duration (Weeks)	Initial assessment	Progression	Muscle/Muscle group targeted	Equipment	Supervised
Toma et al., 2013	Fatigue protocol	Baseline, 24 h and at the 7^th^ day	75% of 1-RM	60 s	1	1-RM	NA	Rectus femoris	Extensor chair	Y
Toma et al., 2016	Strength training	2/week	60–80% 1-RM	Between sets of 2–3 min	8	1-RM	Every 2 weeks (4 sessions): 1-RM again assessed and tailor the load	Knee flex/ext exercise	(Leg Extension SelectionTechnoGym^®^) Biodex System 3 Pro (Biodex Medical System, Shirley, NY, USA)	Y
Matos et al., 2016	Fatigue protocol	Pre-fatigue strength test at 60 s Post-fatigue strength test at 7^th^ minutes from baseline	75% of 1-RM	NA	NA	1-RM	NA	Wrist flexion-extension and hand grip exercises	Sammons Preston Rolyan, Bolingbrook, IL, USA	Y
Vassão et al., 2016	Fatigue protocol	First session, after 7 days second session	Submaximal voluntary concentric muscle contractions	NA	NA	1-RM	NA	Quadriceps and hamstring	BiodexMulti-Joint System 3 isokinetic dynamometer (Biodex Medical System Inc., NY, USA	Y
Vassão et al., 2018	Dynamic strength training program	2	60–80% 1-RM	30 min	8	1-RM	1–2 weeks: 60% 1-MR 2–8 weeks: 80% 1-MR	Quadriceps	Chair (Leg Extension Selection — TechnoGym^®^)	NR
Tucci et al., 2019	Resistance	2	60-80% 1-RM	Between sets rest: 2 min	8	1-RM .	1-MR adjusted at 2, 4, 6, and 8 weeks 1–2 weeks: 60% 1-MR, 2 sets of 15 repetitions 2–8 weeks: 80% 1-MR, three sets of 15 repetitions	Quadriceps	XR-5 leg-extension machine (Righetto Fitness Equipment, Campinas, Brazil)	Y
Fritsch et al., 2019	Resistance	2	NR	Between sets rest: 90–120 s	12	1-RM	1–2 weeks: 2 sets, 18–20 RM 3–4 weeks: 2 sets, 15–17 RM 5–7 weeks: 2 sets, 12–14 RM 8–10 weeks: 3 sets, 8–10 RM 11–12 weeks: 3 sets, 6–8 RM	Quadriceps Other five exercises (seated supine, seated row, leg curl, abdominal curl and back extension exercises)	Biodex System 3 Pro equipment (Biodex Medical Systems, EUA)	NR
Rodrigues et al., 2020	Resistance	3	60% of 1-RM, 3 sets, 10–12 reps	Between sets rest: 2 min	NR	1-RM	Warm-up: 50% load Further: ↑ 5-10%	Quadriceps	Knee extensor machine (Knee Extension; Nakagym^®^, São Paulo, Brazil)	Y
Rodrigues et al., 2022	Resistance	2	50% of 1-RM, 2 sets, 8–12 reps	Between sets rest: 1–2 min	10	1-RM	Every 2 weeks: 2 additional reps, load ↑ 5–10%	Quadriceps	Nakagym equipment, São Paulo, Brazil	Y
Elbanna et al., 2022	Fatigue protocol	3/week	NR	NR	4	NR	2 min intervals, 1 min rest, for 10 min for each foot	Ankle dorsi flexors and plantar flexors	No equipment used	Y

NA Not applicable; NR Not reported; 1-RM one-Repetition Maximum; ↑ increase; Y Yes

**Table 3:** Outline of the main findings of the included studies

Author	Participants characteristics	Outcome measure	Active PBM group		Placebo PBM group		Control group		Results	Conclusion
			Pre	Post	Pre	Post	Pre	Post		
Toma et al., 2013	Participants: Older women Total *N* = 24 2 groups: • Active laser (1^st^ session) and placebo laser (2^nd^ session) *N* = 12 • Placebo laser (1^st^ session) and active laser (2^nd^ session) *N* = 12 Age (Yr): 63.8 ± 2.4	Fatigue protocol: • Repetitions of flexion-extension movement	NR	NR	NR	NR	NA	NA	Order of laser application: No difference among groups (*p* = 0.456) Number of knee flexion-extension repetitions: Placebo Laser: 16.2 (5.91) Active Laser: 18.5 (7.19)	Laser application prior to exercise: ↑ number of knee flexion-extension repetitions
Toma et al., 2016	Participants: Older women Total *N* = 48 3 groups: •CG (*N* = 15) •Strength + placebo LLLT (TG) (*N* = 17) •Strength training + active LLLT (TLG) (*N* = 16) Age (Yr): •CG: 63.64 (± 2.11) •TG: 63.31 (± 2.66) •TLG: 64.07 (± 2.87)	1-RM 6-MWT (m) Isokinetic dynamometry: Peak torque (Nm)	32.5 ± (6.10) 390 60	42.82 ± (7.12) 560 64	31.12 ± (5.05) 400 55	27.71 ± (2.2) 500 56	27.71 ± (2.2) 550 52	27.71 ± (2.2) 560 53	1-RM: Significant ↑ in both TG and TLG in comparison to CG; No difference between TG and TLG Peak torque: Higher in the TGL group compared to CG (*p* = 0.03)	Laser + strength exercises: ↑ 1-RM ↑ peak torque No change in 6-MWT
Matos et al., 2016	Participants: Older women Total *N* = 29 Two groups: •LED group (LG): Active LED (*n* = 15) •Placebo group (PG): Placebo irradiation (*n* = 14)	Fatigue protocol: Grip strength (N): pre and post-fatigue in LG and PG group	217.7 ± 34.3	209.9 ± 35.3	203.0 ± 22.5	181.4 ± 21.6	NA	NA	Significant ↓ in grip strength in PG (*p* < 0.001) No significant difference pre-post in grip strength in the LG group (*p* = 0.063)	Grip strength and reducing muscle fatigue in case of prior application of LED phototherapy
Vassão et al., 2016	Participants: Healthy women Total *N* = 60 •Laser placebo (LP): *n* = 30 •Laser active (LA): *n* = 30) Age (yr): 63.70 (3.02)	Fatigue protocol: Isokinetic dynamometry Electromyographic fatigue index (EFI)	NR	NR	NR	NR	NA	NA	EFI significantly ↑ in the LA group (*p* = 0.011)	PBM application before an isokinetic fatigue protocol attenuated muscle fatigue in elderly women in a single session
Vassão et al., 2018	Participant: Healthy women Total *N* = 35, after drop out *N* = 27 2 groups: • Strength + placebo laser (PG) *N* = 13 • Strength training + active laser (AG) *N* = 14 Age (Yr): • 63.31 ± 2.66 (PG) • 64.07 ± 2.87 (AG)	1-RM (kg) 6-MWT (m) SPPB (Score) Balance: • Fall Risk Test • Postural Stability Test Index (each on Both and Right): • OSI-Both • APSI-Both • MLSI-Both • OSI-Right • APSI-Right • MLSI-Right	36 410 10 1 0.92 ± 0.39 0.69 ± 0.31 0.46 ± 0.29 1.63 ± 0.59 0.98 ± 0.61 0.88 ± 0.52	44 550 11 0.7 0.74 ± 0.27 0.54 ± 0.23 0.38 ± 0.17 1.36 ± 0.96 1.07 ± 0.49 0.54 ± 0.34	30 400 9 1 0.86 ± 0.57 0.59 ± 0.49 0.48 ± 0.27 1.56 ± 0.73 1.10 ± 0.61 1.06 ± 0.51	38 500 10 0.9 0.85 ± 0.35 0.59 ± 0.30 0.46 ± 0.28 1.61 ± 0.78 1.10 ± 0.97 1.02 ± 0.76	NA	NA	1-RM: Intragroup: ↑ 21.7% (PG) ↑ 24.8% (AG) (*p* = 0.001) Between-group: (*p* = 0.633) 6MWT: Intragroup: ↑ 26.6% (PG) ↑ 40.3% (AG) (*p* = 0.001) Between-group: (*p* = 0.175). SPPB: Intragroup: ↑ 0.65% (PG) ↑ 0.91% (AG) (*p* = 0.006) Between-group: (*p* = 0.534) FRT: ↓ AG (*p* = 0.005) PG (*p* = 0.775) PSTI: Intragroup: ↑ MLSI Right (*p* = 0.007) Between-group: (*p* = 0.192)	Exercise program: ↑ Muscle strength ↑ Functional capacity Exercise program + active PBM: ↑ Muscle strength ↑ Functional capacity ↑ MLSI Right ↓ in Fall Risk
Fritsch et al., 2019	Participant: Healthy men Total *N* = 31, after drop out *N* = 24 2 groups: • Strength training + placebo laser (PG) *N* = 13 • Strength training + active laser (PBMT) *N* = 11 Age (Yr): • 66.67 ± 05.84 (PG) • 68.93 ± 07.47 (PBMT)	1-RM (kg) • LP • KE Functional tests • TUG (s) • CRS (s) Peak torque (Nm): • Isometric • Concentric Muscle thickness (cm): • RF MT • VL MT	241.38 (56.43) 88.69 (17.26) 6.38 (0.86) 9.23 (1.26) 191.17 (36.66) 154.95 (27.81) 1.53 (0.27) 196 (0.34)	332.54 (55.18) 108.77 (19.50) 5.96 (0.88) 8.29 (0.94) 201.91 (37.64) 170.45 (28.98) 1.58 (0.28) 2.10 (0.36)	234.18 (27.14) 93.91 (18.08) 6.38 (0.54) 9.78 (0.60) 201.43 (27.70) 168.30 (21.62) 1.54 (0.40) 2.02 (0.28)	326.68 (38.69) 116.82 (23.83) 6.04 (0.32) 8.94 (0.67) 214.40 (26.87) 179.69 (19.97) 1.58 (0.43) 2.15 (0.28)	NA	NA	Mean percent change, ES: PBMT group: LP: 40.00 (14.75), 1.63 KE: 23.43 (12.74), 1.09 TUG: 6.59 (5.98), 0.48 CRS: 9.73 (7.50), 0.85 Isom: 6.22 (8.64), 0.27 Conc: 10.34 (5.19), 0.57 RF: 2.83 (5.35), 0.19 VL: 7.55 (5.91), 0.41 PG group: LP: 40.23 (12.54), 2.81 KE: 24.75 (12.54), 1.11 TUG: 4.59 (6.39), 0.79 CRS: 8.57 (5.68), 1.33 Isom: 6.70 (5.03), 0.48 Conc: 7.14 (6.22), 0.55 RF: 2.64 (3.64), 0.10 VL: 6.74 (8.12), 0.50	Strength training alone and strength training + PBMT: Both groups have similar effects on outcome measures
Tucci et al., 2019	Participants: Older women Total *N* = 42, after drop out *N* = 39 3 groups: • Active PBM + resistance *N* = 12 • Placebo PBM + resistance *N* = 13 • Control group *N* = 14 Age (Yr): • Active group: 65.9 (3.6) • Placebo group: 65.1 (3.5) • Control group: 65.1 (3.5)	1-RM	NR	NR	NR	NR	NR	NR	CG: ↓ in 1-RM over 8 weeks’ time ↑ 1-RM for both PG and AG over 8 weeks’ time	No additional effect of adding the PBM
Rodrigues et al., 2020	Participants: Active older women Total *N* = 17 2 groups: • Active laser *N* = 9 • Placebo laser *N* = 8 After cross over • Active laser (*N* = 8) • Placebo laser (*N* = 9) Age (Yr): 72.6 ± 4.1	SPPB (Score) MVIC (Nm) Endurance: • Repetitions-to-failure test	NR	11.5 ± 0.7 355.3 ± 85.4 11.6 ± 2.2	NR	11.4 ± 1.0 355.1 ± 86.0 11.2 ± 1.9	NA	NA	AG Vs PG: SPPB: No additional effect of PBM, 0.18 mean paired differences, *p* = 0.51 MVIC: No additional effect of PBM, 0.21 mean paired differences, *p* = 0.98 Repetition to failure test: No additional effect of PBM, 0.35 mean paired differences, *p* = 0.30	No additional benefit of using laser in active elderly women
Rodrigues et al., 2022	Participants: Older women Total *N* = 24, after follow up *N* = 22 2 groups: • Leg receiving active laser (*N* = 11) • Leg receiving placebo laser (11) Age (Yr): 66.6 (5.2)	1-RM (kg) Muscle thickness (cm): Vastus lateralis Postural balance • Area of Center of Pressure (cm^2^) • Velocity Anteroposterior (cm/s) • Velocity Mediolateral (cm/s) • Frequency Anteroposterior (Hz) • Frequency Mediolateral (Hz)	28.0 (10.7) 1.86 (0.25) 23.56 (9.55) 4.79 (1.73) 5.61 (1.62) 0.58 (0.21) 0.72 (0.26)	37.0 (13.5) 2.01 (0.27) 17.38 (7.75) 3.94 (1.20) 4.62 (1.14) 0.48 (0.20) 0.62 (0.18)	27.1 (11.5) 1.84 (0.30) 21.77 (12.14) 4.57 (1.88) 5.67 (1.57) 0.64 (0.24) 0.75 (0.22)	36.3 (13.3) 1.96 (0.33) 16.57 (6.78) 3.81 (1.70) 4.75 (1.19) 0.54 (0.18) 0.68 (0.20)	NA	NA	1-RM: Active laser no additional effect Active laser: ↑ VL muscle thickness, 0.58 Effect size Postural variables: Active laser has no additional effect	RT alone: ↑ MT ↑ strength ↑ Postural balance RT + laser: Additional effect on improving MT
Elbanna et al., 2022	Participants: either gender older adults Total *N* = 100 •PBM group (*n* = 50): Age 63.48 ± 2.82 •Placebo group (*n* = 50): Age 63.62 ± 2.87	Fatigability: Fatigue severity scale Function: Katz Index of Activities of Daily Living	4.57 ± 0.26 2.98 ± 0.79	3.97 ± 0.23 3.94 ± 0.77	4.62 ± 0.25 3.04 ± 0.86	4.25 ± 0.26 3.62 ± 0.86	NA	NA	In both the groups significant pre-post difference (*p* < 0.05) within-group comparison Between-group comparison: Significant difference in FSS (*p* < 0.05)	PBM group: ↓ FSS; pre-post 13.13% change Placebo group: ↓ FSS; pre-post 8.1% change

NA: Not applicable; OSI: overall stability index; APSI: anterior/posterior stability index; MLSI: medial/lateral stability index; ES effect size, RF rectus femoris, VL vastus lateralis, MT muscle thickness, Isom isometric, PT peak torque, Isoc isokinetic, LP leg press, 1RM one-repetition maximum, KE knee extension, TUG timed up-and-go, CRS chair rise to standing; A-COP, area of center of pressure; CI, confidence interval; ES, effect size; Freq AP, frequency anteroposterior; Freq ML, frequency mediolateral; RM, repetition maximum; SD, standard deviation; Vel AP, velocity anteroposterior; Vel ML, velocity mediolateral; VL, vastus lateralis

### Risk of bias in individual studies

The risk of bias of the eligible studies was evaluated through the Cochrane Collaboration’s tool for assessing the risk of bias of randomized trials [21]. The classification of this tool includes seven items assessing the risk of bias: selection bias (random sequence generation and allocation concealment), performance bias (blinding of participants and personnel), detection bias (blinding of outcome assessment), attrition bias (incomplete outcome data), reporting bias (selective reporting), and other sources of biases [21]. Two reviewers (PK and GN) critically appraised each of the included studies independently for the risk of bias. A third reviewer (SU) was consulted for consensus rating whenever needed. Items were scored as follows: low risk of bias (+), high risk of bias (-), or unclear risk of bias (?) following the recommended algorithms for reaching the risk-of-bias assessment tool for each domain, as outlined in the respective guidelines [21].

### Strategy for data synthesis

Data from the included studies were extracted and documented in a standard spreadsheet. Qualitative and quantitative data are displayed in tables and summarized in the text to describe the study results of the scoping review questions.

## Results

### Study selection

**Fig. 1:**
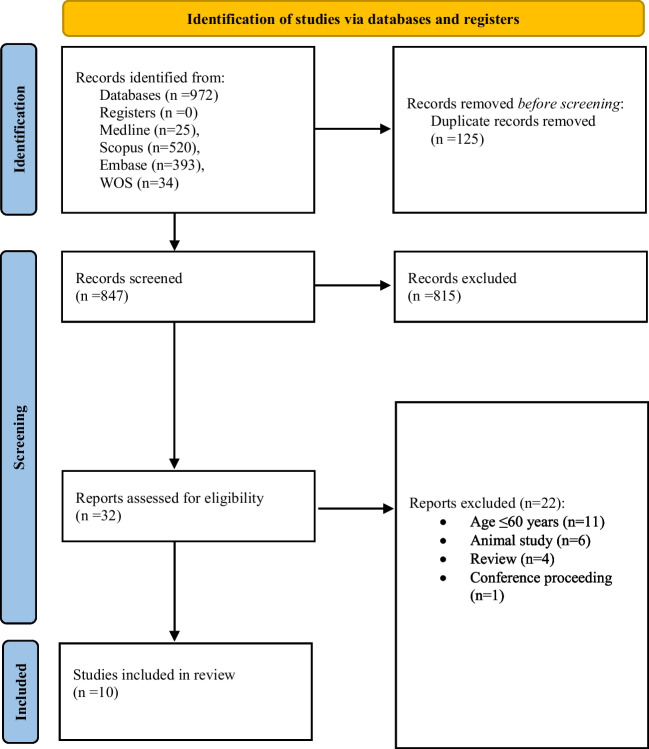
Preferred Reporting Items for Systematic Review and Meta-analysis statement 2020 (PRISMA 2020) flow diagram

A total of 972 articles were identified during the electronic and hand-searching processes, of which 125 were duplicates. After title and abstract screening, the full texts of 32 articles were considered for full text review. Of these, 22 did not meet the inclusion criteria and finally 10 studies were included. A Preferred Reporting Items for Systematic Review and Meta-analysis statement 2020 (PRISMA 2020) [22] flowchart of the literature search is demonstrated in Fig. 1 and PRISMA 2020 expanded checklist as supplementary material 2.

The studies included in this systematic scoping review have a total of 390 older adult (≥ 60 years) participants. Among the 10 studies, eight (*n* = 8, 80%) studies were conducted on older women [23–30], only one (*n* = 1) was performed on older men [31], and one (*n* = 1) included both women and men [32]. The studies included in the review have grouped the participants differently, eight (*n* = 8) studies have divided the participants into two groups (active laser/ placebo laser) [23, 25–28, 30–32], and two (*n* = 2) have three participant groups (active laser/ placebo laser/ control) [24, 29]. Five (*n* = 5) studies are, placebo-controlled, double blind randomized controlled trial [24, 26, 30–32], one (*n* = 1) is a randomized, triple blinded, placebo controlled study [28], three (*n* = 3) is randomized, double blinded, crossover trial with placebo controlled [23, 25, 27], and one (*n* = 1) is three arm, parallel, randomized controlled trial [29].

### Descriptive details of the dosimetry of the photobiomodulation therapy used in the studies

 The PBM was given as LASER in nine (*n* = 9) studies [23–29, 31, 32], and only one (*n* = 1) used a light emitting diode (LED) [30]. Continuous mode has been used in all the included studies. The peak power of 100 mW has been used in nine (*n* = 9) of the studies [23–29, 31, 32], with only one (*n* = 1) have used 150 mW as peak power [30]. The LASER wavelength ranged from 808 nm to 850 nm, with eight studies (*n* = 8, 80%) have used 808 nm wavelength [23–29, 32], and only one (*n* = 1) have used 850 nm wavelength [31]. One study have utilized LED with a wavelength of 638 nm [30]. The power density ranges from 2 W/cm^2^ to 35.7 W/cm^2^. Five (*n* = 5) studies have used 35.7 W/cm^2^ [24–28], one (*n* = 1) each has used very low power of 2 W/cm^2^ [29], 3.4 W/cm^2^ [31], and 12.7 W/cm^2^ [23]. The two (*n* = 2) studies have not reported the power density [30, 32]. Energy density ranges from 11.7 J/cm^2^ to 892 J/cm^2^. The common most energy density used in five (*n* = 5) studies is 250 J/cm^2^ [24–28], one (*n* = 1) study each used 11.7 J/cm^2^ [30], 91 J/cm^2^ [29], 127.39 J/cm^2^ [32], 206.9 J/cm^2^ [31], and 892 J/cm^2^ [23]. The muscle group targeted in the eight (*n* = 8, 80%) of the included studies is the knee extensor group, with the belly of the rectus femoris muscle as the site of the delivery [23–29, 31]. In one (*n* = 1) study each, the upper limb forearm muscle group [30] and lower limb soleus muscle group have been used [32]. In the majority of the studies (*n* = 6, 60%), PBM therapy was delivered to eight sites [23–27, 31], followed by two (*n* = 2) studies have used 4 sites [29, 30], one (*n* = 1) each study have used 6 sites [28], and 2 sites [32]. The energy of 7 J is most commonly delivered per site in six (*n* = 6) studies [23–28]. One (*n* = 1) study each delivered an energy of 4 J [29], 4.5 J [30], and 30 J [31] per site, and one (*n* = 1) did not report the energy delivered per site [32]. Duration per site ranges from 20 to 70 s, with six (*n* = 6) studies delivering for 70 sec [23–28], two (*n* = 2) for 40 sec [29, 32], and one (*n* = 1) each for 20 sec [30] and 60 sec [31]. Per diode, spot size ranges from 0.00785 to 0.05, with the most common being 0.028 cm^2^ in five (*n* = 5) studies [24–28]. The stationary and skin contact was the most commonly used method of delivery used in seven (*n* = 7) studies [23, 24, 27–31], with three (*n* = 3) studies having used punctual contact [25, 26, 32]. Delivery of the photobiomodulation was done before the exercise training session in six (*n* = 6) studies [23, 25, 27, 28, 30, 31], while post-exercise session delivery was used in three (*n* = 3) studies [24, 26, 29], and in one (*n* = 1) study it was delivered simultaneously while performing the repetitive ankle dorsi and plantar flexion [32]. The details of the parameters of PBM therapy are provided in Table 1.

### Components of exercise training sessions delivered in the studies

We have described the results of the exercise training sessions in the format of the FITT (frequency, intensity, time, and type) for easier understanding of medical professionals and rehabilitation experts. The resistance/ strength training is the most common type of training used in six (*n* = 6) studies [24, 26–29, 31], with four (*n* = 4) of the studies have used the fatigue protocol [23, 25, 30, 32]. The frequency per week ranges from 2 to 3 times per week, with five (*n* = 5) studies delivered it 2 times per week [24, 26, 28, 29, 31]. The intensity of the resistance training was decided using the one-Repetition Maximum (1-RM). The intensity used is moderate to high intensity, ranging from 50% of 1-RM to 80% of 1-RM. Three (*n* = 3) studies have used 60-80% of 1-RM intensity [24, 26, 29]. One study has not reported the intensity [31], with one each has used 50% [28], and 60% [27] of 1-RM as intensity. The exercise sets and number of repetitions range from 2 to 3 sets with 8–12 repetitions. The progression of the training was done based on increasing the percentage of 1-RM (5-10%) [26–29] or the number of sets and reps per set. The total time per session of resistance training has been mentioned only in one (*n* = 1) study of 30 min [26], rest studies have mentioned the 2–3 min of rest period provided between sets [24, 27–29, 31]. Among the muscle groups used for resistance training, the knee extensor (quadriceps) muscle group is the commonest one [23, 24, 26–29, 31]. The total duration of the exercise session ranges from 8 to 12 weeks. Three (*n* = 3) studies have delivered the exercise training for 8 weeks [24, 26, 29], one (*n* = 1) each for 1 week [23], 4 weeks [32], 10 weeks [28] and 12 weeks [31], with three (*n* = 3) have not reported the number of weeks exercise delivered for [25, 27, 30]. To find out one of the important things while delivering the exercise session to older adults, which is supervision, the exercise session delivered was supervised in eight (*n* = 8) studies, while two (*n* = 2) have not reported [26, 31]. The description of the components of resistance exercises can be found in Table 2.

### Effect of photobiomodulation as an adjunct to the resistance training on muscle metrics

Seven (*n* = 7) of the ten studies included in the review have assessed the 1-RM. Out of these seven studies, two (*n* = 2) studies have used 1-RM to decide the intensity of fatigue protocol and not as an outcome measure. Three studies have two groups [26, 28, 31], one active PBM group and other placebo PBM group. There found to be increase in the 1-RM in all the studies in both the groups, with no significant difference between the groups. The two (*n* = 2) studies [24, 29] having three groups (active PBM, placebo PBM, and control group) have found that when compared to the control group, there was a significant increase in the 1-RM in active and placebo PBM groups. However, no difference was found between the PBM groups. The second component of muscle metric, peak torque (nm), has been used as the outcome measure in three (*n* = 3) studies [24, 27, 31]. An increase in the peak torque within group in both the active and placebo PBM therapy was reported. However, between group comparisons have found no additional benefit of adding PBM. The third component of muscle metrics, muscle thickness, has been used as an outcome measure in two (*n* = 2) studies [28, 31]. Muscle thickness is an important outcome measure, especially in research related to older adults who have a poverty of muscle, sarcopenia. Both the studies which have utilized muscle thickness as an outcome measure have measured the thickness of a large lower limb muscle group, with the commonest muscle being the vastus lateralis and rectus femoris of knee extensor groups. The results of the study suggest that resistance exercises alone can improve muscle thickness, with one study found that the addition of PBM has an additional effect in improving muscle thickness [28].

### Effect of photobiomodulation as an adjunct to the resistance training on functional balance

Three (*n* = 3) studies out of the seven studies have considered balance as an outcome measure [26, 28, 31]. The three studies utilised different tests to assess the balance with one (*n* = 1) have used a functional test like timed-up-go and chair rise to standing [31], one (*n* = 1) used fall risk test and functional stability test [26], one (*n* = 1) have used analysis of center of pressure to calculate the following balance parameters: area of center of pressure (A-COP), velocity anteroposterior (Vel AP), velocity mediolateral (Vel ML), frequency anteroposterior (Freq AP), and frequency mediolateral (Freq ML) [28]. Since all the studies have used different tests to assess the balance, a definite conclusion cannot be made. However, one of the studies found improvement in the balance of the participants in the active PBM group, which received both resistance and active LASER [26], with two (*n* = 2) stating similar effects between the active and placebo groups [28, 31].

### Effect of photobiomodulation as an adjunct to the resistance training on functional capacity and physical performance.

Two (*n* = 2) studies have assessed the functional capacity of older adults using the six-minute walk test (6-MWT). One study with three groups (active PBM/ placebo PBM/ control group) suggested that between group comparison, there was no significant differences in the functional capacity [24]. In the second study with active and placebo PBM groups, there found to be an increase in the functional capacity by 40.3% in the active and 26.6% in the placebo group with a p-value of 0.001 intragroup comparison, however, this difference between the groups was not statistically significant (p-value 0.175) [26]. The physical performance of older adults has been assessed in two (*n* = 2) studies using the Short Physical Performance Battery (SPPB). Intragroup comparison has suggested an increase in both the groups, with 0.91% and 0.65% increase in SPPB score in active and placebo groups, respectively [26]. However, the between group comparison was not significant, with a p-value of 0.534. Similarly, in the second study there found to be no additional effect of using PBM on the physical performance of the older adults [27].

### Effect of photobiomodulation on the fatigability

Four (*n* = 4) studies have assessed the aspect of fatigability among older adults having received the PBM [23, 25, 30, 32]. Of the three studies, one (*n* = 1) used LED [30] and three (*n* = 3) have used LASER [23, 25, 32]. The participants received the phototherapy before the fatigue protocol in three (*n* = 3) studies [23, 25, 30], and simultaneously along with the protocol in one (*n* = 1) study [32]. The findings support the prior application of PBM in reducing fatigability [23, 25, 30, 32]. The study participants were older women in three (*n* = 3) studies [23, 25, 30] and one (*n* = 1) study had both the genders [32]. There found an increased number of repetitions of knee flexion-extension [23] and no change in pre-post grip strength (p-value 0.063) following the fatigue protocol in the active PBM therapy group when compared to the placebo group [30]. The study utilized the Electromyographic Fatigue Index (EFI) have reported a significant increase in EFI in the active LASER group (p-value 0.011) compared to the placebo LASER group [25]. Lastly, the study conducted on either gender reported a pre-post decrease of 13.13% in the Fatigue Severity Scale (FSS) in the active PBM group as compared to a change of 8.1% in the placebo PBM group (p-value < 0.05) [32].

The outline of the main findings of the included studies with active PBM therapy group details, placebo PBM therapy group details for outcomes muscle metrics, functional balance, functional capacity, physical performance, and fatigability are provided in Table 3.

### Risk of bias assessment

**Fig. 2:**
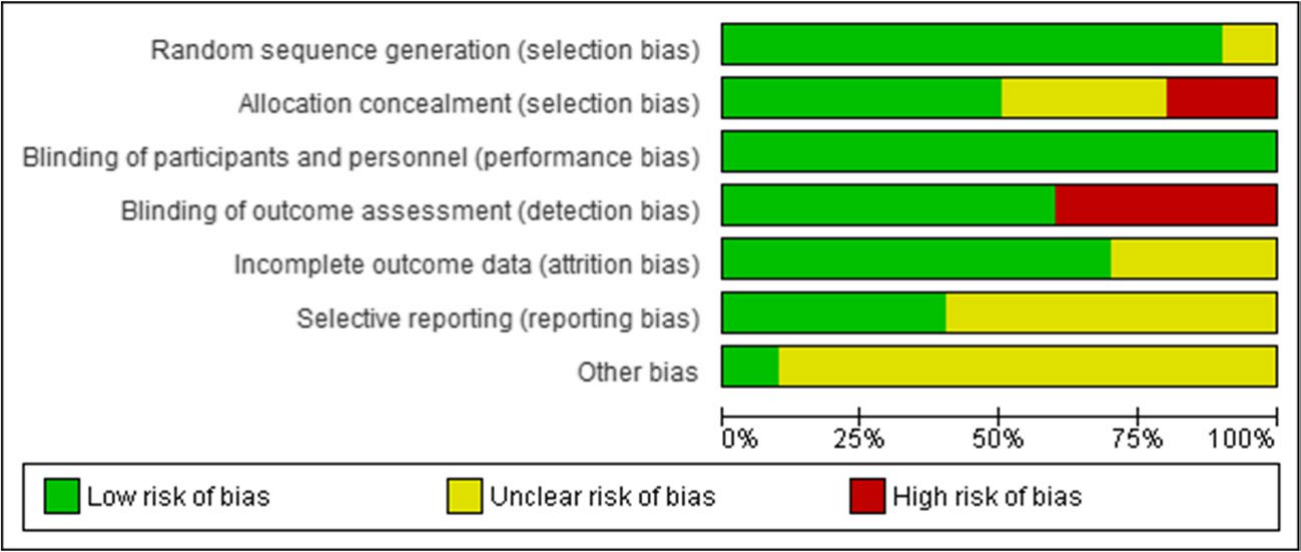
Risk of bias graph

**Fig. 3:**
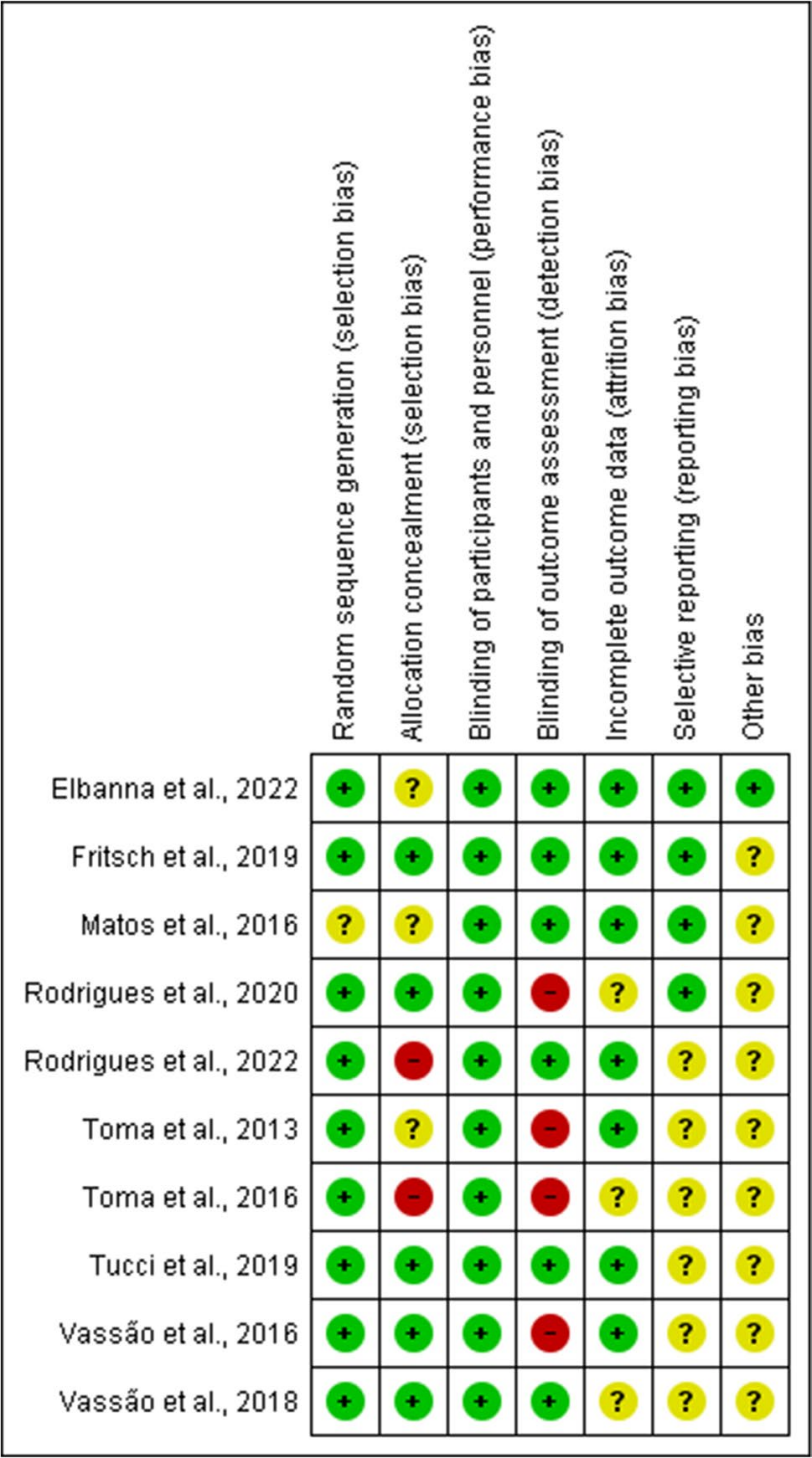
Risk of bias summary

The risk of bias analysis demonstrated a low risk of bias for random sequence generation, with most studies (90%, *n* = 9) providing clear information. The analysis for allocation concealment suggests *n* = 5, 50% of the study with low risk, with the remaining *n* = 5 divided as an unclear risk 30%, *n* = 3; high risk *n* = 2, 20%. The analysis revealed no lack of performance bias, however there found to be a detection bias in (40%, *n* = 4) studies. The details of the risk of bias assessment of all included studies are summarized in Figs. 2 and 3.

## Discussion

This review has scoped the parameters of PBM, and resistance exercise used among older adults. Additionally, the review has descriptively summarized the change in muscle metrics, functional balance, functional capacity, physical performance, and fatigability observed following the application of PBM and/ or resistance exercise.

The review found that LASER is the most commonly used tool to deliver phototherapy compared to LED. There exists a variation in the studies regarding the time of application of PBM, either before the resistance training or after. The review found that six (*n* = 6) studies out of the included ten studies applied the PBM before the training. In 2016, Vanin and colleagues conducted a study aimed at identifying the most effective timing for administering phototherapy irradiation in conjunction with strength training. The study found that administering active phototherapy prior to resistance training, followed by a placebo treatment afterwards, yielded positive results [10]. Administering PBM prior to resistance exercise is found to reduce post-training fatigue.

This review found a range of LASER wavelength from 808 nm to 850 nm used, which is similar to the findings suggesting the optimum wavelength near to 810–840 nm, since in these regions the surface chromophores have weak absorption, and therefore there is maximum penetration of light into the skin, generating an optimal window of penetration and absorption by organic molecules [33]. The majority of studies (*n* = 8, 80%) included in this review utilized a wavelength of 808 nm. For young healthy adults, recent evidence has recommended a wavelength of 640 nm (red) to 950 nm (infrared) [17, 18]. There found to be an added benefit of employing PBM therapy alongside resistance exercise compared to the control group. However, the outcomes did not show significant variance between the active LASER and placebo LASER groups. A recent systematic review and meta-analysis identified a wavelength range of 655 nm to 905 nm for healthy individuals [34]. This indicates that PBM therapy applications could be tailored to different age groups to address their unique physiological needs. The 808 to 850 nm range is commonly used for its ability to penetrate deeper into tissues. This near-infrared wavelength effectively targets muscles, joints, and deeper cellular structures, stimulating mitochondrial activity, boosting ATP production, and supporting cellular repair and regeneration. This makes it particularly beneficial for older adults. On the other hand, younger adults, including athletes, benefit from a wider range of wavelengths, typically from 655 to 905 nm. This spectrum includes both red light (around 655 nm) and near-infrared light (up to 905 nm), each offering its own advantages. Therefore, the specific physiological challenges faced by each age group underpin the scientific rationale for these different wavelength preferences.

Utilizing resistance training as a mode of exercise intervention is a common practice among older adults seeking to enhance their muscle metrics [7]. Interestingly, the studies included in this review reveal similarities, with resistance exercise being employed along with PBM therapy as the most common mode of exercise intervention. The delivery of the resistance exercises in the included studies has followed the frequency, intensity, time, and type framework. The components of resistance exercise intervention used in the studies included in this review are in line with the resistance exercise parameters used in the management of sarcopenic older adults [7, 35]. The studies in the review have used the leg extension machine system to perform the resistance training of the major muscle group of the lower limb, quadriceps. However, these extension machines have a demerit of measuring a single joint movement, which does not reflect the muscle power required while doing functional activities like a chair rise which involve multi-joint leg extension [36, 37].

The rising population of older adults globally is a serious concern with a parallel increase in the prevalence of a common geriatric syndrome, sarcopenia [38]. The decrease in muscle strength is a prominent feature among older adults. This review found that the PBM have a significant effect on improving the 1-RM among older adults when compared to the control group. However, there found to be no significant difference in muscle strength between the active PBM and placebo PBM. Similar findings have been reported in the study conducted on post-menopausal women which shows that PBM was not able to induce additional benefits to resistance training to improve muscle strength [39]. In this review, we found that there was an increase in the peak torque pre-post when compared within the group, with no significant difference between the active and placebo groups. The study conducted on young adult males found an increase in the peak torque if the PBM is applied before the resistance training [10]. A study on male Wistar rats by Albuquerque-Pontes et al., 2015 has reported that the irradiation of healthy muscles by phototherapy leads to increased cytochrome c-oxidase activity. The study utilized different doses (660 nm, 830 nm, 905 nm) to irradiate the tibialis anterior muscle and reported benefits with PBM therapy doses of 660 nm with 1 J, 808 nm with 3 J, and 905 with 1 J, protecting against skeletal muscle tissue damage and enhancing performance [40]. This review also reports the most common wavelength of 808 nm (near infrared) used for older adults. This may partially explain the mechanism, however human studies on cytochrome activity are limited.

The impact of PBM therapy on the functional balance of older adults is not yet fully understood. Only three studies have used balance as an outcome measure, which limits our understanding of its effects. The two studies reported no additional benefit of PBM therapy along with resistance exercise as compared to resistance exercise alone. One study reported an increase in stability and a decrease in fall risk among active LASER group participants. Additionally, the wide range of tests used to assess balance further complicates drawing clear conclusions about the benefits of PBM therapy. Improvement in strength and physical performance could be the main reason alongside PBM may have an influence on the sensory inputs specifically proprioception thereby improving functional balance, however these mechanisms need further confirmatory studies.

Among older adults, the decline in functional capacity and physical performance is a serious concern, often resulting from muscle atrophy, reduced mitochondrial function, and increased inflammation. The application of PBM therapy could have a positive effect, considering that PBM improves microcirculation, enhancing oxygen and nutrient delivery to muscles during and after exercise [41]. The combined effects of increased ATP production, enhanced muscle protein synthesis, and improved circulation could result in significant improvement in functional capacity and physical performance among older adults. This review found only two (*n* = 2) studies have used functional capacity and physical performance as one of the outcome measures, indicating a new area that would require further research to get more clarity on the use of the application of PBM therapy as an adjunct to resistance exercise. The studies included in this review reported no additional benefits of adding the PBM therapy along with resistance exercise on functional capacity and physical performance. Similar results have been reported in a systematic review and meta-analysis assessing the effect of PBM therapy in healthy individuals [34]. Another study done on young healthy women reported no enhancement in muscle performance following PBM therapy [42].

The studies in this review which have explored the effect on the level of fatigue, three of the studies were conducted on healthy older women, while one on either gender. The findings of the study included in this review provide evidence in support of the application of PBM to reduce the level of fatigue among older adults. A recently published systematic review with meta-analysis also supports the evidence about the PBM reducing fatigue [34]. Delayed onset of fatigue following the application of PBM therapy could be through several potential mechanisms. PBM therapy enhances the mitochondrial function by stimulating the cytochrome c oxidase, a key enzyme in the mitochondrial electron transport chain. This stimulation boosts ATP synthesis, providing cells with more energy to function effectively [41]. Additionally, PBM therapy reduces oxidative stress and inflammation, which are common contributors to fatigue in older adults, thus alleviating muscle soreness and overall muscle function, thereby delaying the onset of fatigue [43, 44]. A study conducted on young women has reported reduced levels of fatigue following the application of PBM [45]. Another study conducted on young women found no significant difference between active LLLT and placebo group on muscle fatigue, although a tendency was observed in the active laser group toward showing lower electromyography fatigue of the biceps brachii muscle [46]. Another study conducted on male athletes has reported the ergogenic effect of the PBM evident from the delayed onset of fatigue [47], although similar mechanisms are documented, it may not be comparable with older adults since athletes are trained for high performance.

When incorporating PBM therapy as an adjunct to resistance training, it is crucial to consider the dosimetry parameters involved in order to achieve the intended therapeutic effect. These parameters determine the amount of light energy delivered to the target tissue and significantly impact the clinical outcomes of PBM therapy. To better understand PBM therapy dosimetry, healthcare professionals can refer to Table 4 for a comprehensive summary of the various parameters involved in targeting specific outcome measures. Adjusting treatment parameters with this information can optimize the effectiveness of PBM therapy and improve treatment outcomes for patients.

**Table 4:** Summary of the PBM therapy dosimetry for each outcome measure

Outcome	Wavelength	Peak power (mW)	Power density (W/cm^2^)	Energy density (J/cm2)	Energy per site (J)	Energy per leg (J)	Treatment time (sec/site)	Spot size (cm^2^)	Application
1-RM	808	100	2-35.7	91–250	4–30	42–240	40–70	0.028–0.05	Post-exercise session
Peak torque	808–850	100	3.4–35.7	206.9–250	7–30	56–240	60–70	0.028–0.029	Pre-exercise session
Muscle thickness	808–850	100	3.4–35.7	206.9–250	7–30	42–240	60–70	0.028–0.029	Pre-exercise session
Functional balance	808–850	100	3.4–35.7	206.9–250	7–30	42–240	60–70	0.028–0.029	Pre-exercise session
Functional capacity	808	100	35.7	250	7	56	70	0.028	Post-exercise session
Functional performance	808	100	35.7	250	7	56	70	0.028	Pre or Post-exercise session
Fatigability	638–808	100–150	12.7–35.7	11.7–892	4.5-7	56	20–70	0.0078–0.038	Before fatigue protocol

Strengths and limitations: First, a comprehensive systematic search strategy was performed in four electronic databases, to identify a broad range of studies related to the topic. Second, no time restriction in the search strategy strengthens the search for literature published on the topic. Third, we followed acknowledged method recommendations for scoping reviews and did duplicate study selection and data extraction to raise validity. Also, to the best of our knowledge, this is the first study systematically scoping the literature regarding the application of PBM therapy and its dosimetry, components of resistance exercises, and the effect of PBM as an adjunct to resistance exercise. Lastly, we have used the risk of bias 2.0 tool to add value to the evidence provided in this review.

This study has a few limitations as well. First and foremost, majority of the studies were conducted among older women, which limits the generalisation of the review findings and thus requires caution in interpreting the results. Second, the findings of the review lack objective meta-analysis evidence, as the purpose of this systematic scoping review was to find out and explore the existing studies’ details on the use of PBM therapy as an adjunct to resistance exercises among older adults. Third, a review has considered only the published articles in electronic databases. Fourth, only full-text articles were considered as abstracts, and proceedings were excluded. Fifth, we did not use search strategies with terms other than English, and we may have missed eligible studies in the other languages we intended to include, which could have enriched the review.

Future recommendations: Conducting future meta-analyses could provide a more robust quantitative understanding of the efficacy of photobiomodulation therapy as an adjunct to resistance exercise. Furthermore, to improve the quality and applicability of results, future research should focus on increasing the number of studies, standardizing protocols and including rigorous quantitative analysis of the data. Including studies in multiple languages and considering currently unextractable data could also enrich the understanding of the impact of PBM.

## Conclusion

The findings of this systematic scoping review underscore the potential of photobiomodulation (PBM) as an adjunct to resistance exercise in enhancing muscle metrics, functional balance, capacity, and performance among older adults. While PBM, particularly delivered via LASER with wavelengths ranging from 808 to 850 nm, shows promise in reducing fatigue and supporting muscle function, the evidence does not consistently demonstrate significant additional benefits over resistance exercise alone. The variability in dosimetric parameters and study designs highlights the need for standardized protocols to optimize the application of PBM. Future research should focus on establishing clear guidelines for PBM dosimetry and exploring its long-term effects on sarcopenia management in older populations. This review provides a valuable foundation for clinicians and researchers aiming to integrate PBM into therapeutic regimes for ageing individuals, promoting better health outcomes through combined physical and phototherapeutic interventions.

## Supplementary information

Below is the link to the electronic supplementary material.

Supplementary file1 (DOCX 16.0 KB)

Supplementary file2 (DOCX 40.7 KB)
