# Supplementary material for: Photobiomodulation therapy as an adjunct to resistance exercises on muscle metrics, functional balance, functional capacity, and physical performance among older adults: A systematic scoping review
Source: Lasers Med Sci. 2024 Sep 3;39(1):232. doi: 10.1007/s10103-024-04177-x (PMC11371873; doi:10.1007/s10103-024-04177-x)
Supplement: Supplementary file 1 — Supplementary file1 (DOCX 16.0 KB) [file 10103_2024_4177_MOESM1_ESM.docx]

Supplementary material 1: Search strategy

Medline

| #1 | "phototherapy"[All Fields] OR "photobiomodulation"[All Fields] OR "light emitting diode"[All Fields] OR "LASER therapy"[All Fields] | 77,415 |
| --- | --- | --- |
| #2 | "resistance training"[All Fields] OR "strengthening exercise"[All Fields] OR "exercise"[All Fields] | 491,233 |
| #3 | "older adults"[All Fields] OR "elderly"[All Fields] OR "older men"[All Fields] OR "older women"[All Fields] | 444,637 |
| #4 | ("phototherapy"[All Fields] OR "photobiomodulation"[All Fields] OR "light emitting diode"[All Fields] OR "LASER therapy"[All Fields]) AND ("resistance training"[All Fields] OR "strengthening exercise"[All Fields] OR "exercise"[All Fields]) | 977 |
| #5 | ("phototherapy"[All Fields] OR "photobiomodulation"[All Fields] OR "light emitting diode"[All Fields] OR "LASER therapy"[All Fields]) AND ("resistance training"[All Fields] OR "strengthening exercise"[All Fields] OR "exercise"[All Fields]) AND ("older adults"[All Fields] OR "elderly"[All Fields] OR "older men"[All Fields] OR "older women"[All Fields]) | 25 |

Embase

| #1 | 'phototherapy'/exp OR 'phototherapy' OR 'photobiomodulation'/exp OR 'photobiomodulation' OR 'low level laser therapy'/exp OR 'low level laser therapy' | 123,557 |
| --- | --- | --- |
| #2 | 'resistance training' OR 'strengthening exercise' OR 'exercise' | 716,551 |
| #3 | 'older adults' OR 'older people' OR 'aged' OR 'geriatrics' | 6,037,783 |
| #4 | #1 AND #2 | 1,827 |
| #5 | #4 AND #3 | 393 |

Web of science

| #1 | (((ALL=("phototherapy")) OR ALL=("photobiomodulation")) OR ALL=("low level laser therapy")) OR ALL=("LASER therapy") | 31,213 |
| --- | --- | --- |
| #2 | (((ALL=("strengthening exercise")) OR ALL=("strength training")) OR ALL=("Resistance exercise")) OR ALL=("exercise") | 657,384 |
| #3 | (((ALL=("older adult")) OR ALL=("elderly")) OR ALL=("older men")) OR ALL=("older person") | 414,860 |
| #4 | #1 AND #2 | 950 |
| #5 | #4 AND #3 | 34 |

Scopus

| #1 | ( TITLE-ABS-KEY ( photobiomodulation ) OR TITLE-ABS-KEY ( laser ) OR TITLE-ABS-KEY ( phototherapy ) ) | 1,633,852 |
| --- | --- | --- |
| #2 | ( TITLE ( elderly ) OR TITLE ( elderly AND women ) OR TITLE ( elderly AND men ) ) | 179,326 results |
| #3 | ( ( TITLE ( elderly ) OR TITLE ( elderly AND women ) OR TITLE ( elderly AND men ) ) ) AND ( ( TITLE-ABS-KEY ( photobiomodulation ) OR TITLE-ABS-KEY ( laser ) OR TITLE-ABS-KEY ( phototherapy ) ) ) | 520  [520 results](https://www.scopus.com/search/history/results.uri?origin=searchhistory&shid=17) |
